# When Defenses Fail: *Atelopus zeteki* Skin Secretions Increase Growth of the Pathogen *Batrachochytrium dendrobatidis*

**DOI:** 10.1093/icb/icac060

**Published:** 2022-05-31

**Authors:** Jordan Gass, Jamie Voyles

**Affiliations:** Department of Biology, University of Nevada at Reno, 1664 North Virginia Street, Reno, NV 89557, USA; Department of Biology, University of Nevada at Reno, 1664 North Virginia Street, Reno, NV 89557, USA

## Abstract

To combat the threat of emerging infectious diseases in wildlife, ecoimmunologists seek to understand the complex interactions among pathogens, their hosts, and their shared environments. The cutaneous fungal pathogen *Batrachochytrium dendrobatidis* (*Bd*), has led to the decline of innumerable amphibian species, including the Panamanian golden frog (*Atelopus zeteki*). Given that *Bd* can evade or dampen the acquired immune responses of some amphibians, nonspecific immune defenses are thought to be especially important for amphibian defenses against *Bd*. In particular, skin secretions constitute a vital component of amphibian innate immunity against skin infections, but their role in protecting *A. zeteki* from *Bd* is unknown. We investigated the importance of this innate immune component by reducing the skin secretions from *A. zeteki* and evaluating their effectiveness against *Bd in vitro* and *in vivo*. Following exposure to *Bd* in a controlled inoculation experiment, we compared key disease characteristics (e.g., changes in body condition, prevalence, pathogen loads, and survival) among groups of frogs that had their skin secretions reduced and control frogs that maintained their skin secretions. Surprisingly, we found that the skin secretions collected from *A. zeteki* increased *Bd* growth *in vitro*. This finding was further supported by infection and survival patterns in the *in vivo* experiment where frogs with reduced skin secretions tended to have lower pathogen loads and survive longer compared to frogs that maintained their secretions. These results suggest that the skin secretions of *A. zeteki* are not only ineffective at inhibiting *Bd* but may enhance *Bd* growth, possibly leading to greater severity of disease and higher mortality in this highly vulnerable species. These results differ from those of previous studies in other amphibian host species that suggest that skin secretions are a key defense in protecting amphibians from developing severe chytridiomycosis. Therefore, we suggest that the importance of immune components cannot be generalized across all amphibian species or over time. Moreover, the finding that skin secretions may be enhancing *Bd* growth emphasizes the importance of investigating these immune components in detail, especially for species that are a conservation priority.

## Introduction

Emerging infectious diseases (EIDs) are one of the leading causes of biodiversity loss, contributing to the Earth's sixth mass extinction (Wake and Vredenburg 2008;Cunningham et al. 2017). To decipher the causes and consequences of wildlife diseases, the field of ecoimmunology takes a holistic approach to understanding complex host–pathogen interactions in the context of their shared environment (Schoenle, Downs, and Martin 2018). Ecoimmunologists seek to uncover the components and mechanisms of host immune systems, even though it can be difficult to pinpoint precise immune mechanisms underlying host susceptibility or resistance in the context of a severe and rapidly spreading disease (Lafonte and Johnson 2013). This challenge is particularly arduous when there is a high degree of interspecific variation in immune defense mechanisms against a generalist pathogen. What “works” for one host species (a defense that reduces pathogen burdens and alleviates disease) may be ineffective—or even detrimental—for another. Although disentangling these complexities is challenging, it is also integral to understanding the diverse range of host responses to outbreaks (i.e., epizootics) and for generating science-based management strategies to mitigate threats to the most vulnerable species.

A compelling vertebrate taxon for exploring diverse host responses to EIDs is amphibians (Skerratt et al. 2007). Amphibians are experiencing precipitous declines due to the emergence of the fungal pathogen, *Batrachochytrium dendrobatidis* (*Bd*), which causes the disease chytridiomycosis (Berger et al. 1998; Longcore et al. 1999; Scheele et al. 2019). One recent assessment suggests that *Bd* has now led to declines in at least 501 amphibian species and driven an estimated 90 of these species to presumed extinction (Scheele et al. 2019). Variation in pathogenicity among *Bd* strains as well as interspecific differences in host susceptibility makes resolving the complex mechanisms behind disease development challenging (Dang et al. 2017). While amphibians have complex acquired (pathogen specific) and innate (nonspecific) immune responses, *Bd* may evade or dampen acquired immune responses (Rosenblum et al. 2009; Fites et al. 2013). For example, there is evidence that *Bd* inhibits lymphocyte gene expression in *Atelopus zeteki* (Ellison et al. 2014), as well as lymphocyte proliferation in *Xenopus laevis* and *Lithobates* (*Rana*) *pipiens* (Fites et al. 2013), suggesting that cell-mediated immune responses against *Bd* infection may not be effective. With evidence that the acquired immune system is relatively ineffective against *Bd*, many investigators have instead focused on the amphibian innate immune system and found encouraging evidence that some aspects of non-specific defenses may be effective in defending amphibian hosts against *Bd* infection, disease development, and mortality (Rollins-Smith et al. 2005; Woodhams et al. 2006; Woodhams et al. 2010).

One aspect of the innate immune system of amphibians is their skin secretions, which include the cutaneous microbiome, antimicrobial peptides (AMPs), antibodies, lysozyme, toxic alkaloid compounds, as well as proteins, carbohydrates, and other chemically rich compounds (Varga et al. 2019, reviewed in Grogan et al. 2020). Of these components, the inhibitory effects of AMPs against *Bd* have been well documented across a broad range of amphibian species (Woodhams et al. 2006; Rollins-Smith 2009). AMPs are small cationic peptides that are secreted from granular glands on amphibian skin (Rollins-Smith et al. 2005) and can degrade a wide diversity of pathogens, including viruses, bacteria, protozoa, and fungi (reviewed in Nicolas and Mor 1995). Many past studies provide strong evidence that AMPs play an essential role in protecting against *Bd* pathogenesis (Ramsey et al. 2010; Grogan et al. 2018). However, the diversity and quantity of AMPs differ among amphibian species (reviewed by Varga et al. 2019). It is generally thought that variation among AMP repertoires underpins interspecific differences in host responses to *Bd* infection and therefore, explains differences in resistance and susceptibility among amphibian species (Li et al. 2007; Woodhams et al. 2010).

**Fig. 1:**
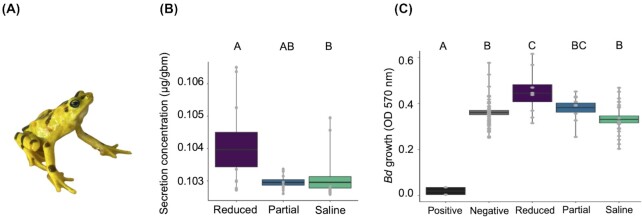
(**A**) A Panamanian golden frog (*A. zeteki*). (**B**) Mean concentrations [μg/gram body mass (gbm)] of skin secretions recovered from *A. zeteki* following injections with norepinephrine (NE) at two different doses [40 nmol/gbm for the “reduced” group (purple) or 10 nmol/gbm for the “partially reduced” (blue)] or sterile saline (teal). (**C**) Viability of *Bd* in natural mixtures of skin secretions collected from *Lithobates pipiens* (positive control, black), sterile amphibian phosphate buffered saline (aPBS, negative control, grey), natural mixtures of skin secretions collected from *A. zeteki* in the reduced treatment group (purple), the partially reduced treatment group (blue), the saline groups (teal). *Bd* viability was measured on the day of peak growth as optical density (OD 570 nm). Error bars indicate standard error of the mean. Letters indicate significant differences among groups at the level of *P* = 0.05.

One host species that is highly susceptible to chytridiomycosis is the Panamanian golden frog *Atelopus zeteki* (Fig. 1A). Following the emergence of *Bd* in Central America, many populations of *A. zeteki* experienced massive *Bd* induced declines and the species was listed as critically endangered (Lips et al. 2006; Crawford et al. 2010; McCaffery et al. 2015; IUCN Red List 2021). To ensure the survival of this species, the captive breeding initiative “Project Golden Frog” was established prior to *Bd* spread in Panama (Gagliardo et al. 2008; Poole 2008). Because *Bd* is still present and highly pathogenic in Panama (Perez et al. 2014; Voyles et al. 2018; Rosa et al., in press), ​conservation efforts have shifted towards understanding *Bd–Atelopus* interactions, using animals from captive populations with the goal of returning this species to the wild (Lewis et al. 2019). Previous studies on *A. varius*, a species closely related to *A. zeteki* that also experienced *Bd* induced declines in Panama (Voyles et al. 2018; Byrne et al. 2021), suggested that skin secretion samples collected from *A. varius* in the wild contained fewer AMPs compared to more resistant species (Woodhams et al. 2006). In addition, a recent study revealed that the AMPs of wild *A. varius* had greater anti-*Bd* properties compared to those captive *A. varius* (Voyles et al. 2018). These previous studies prompted our investigation into the importance of skin secretions in protecting captive *A. zeteki*. Given the complex interactions between the amphibian innate immune system and *Bd, in vivo* experiments are essential to understanding the role of skin secretions in disease development for this highly vulnerable species.

The purpose of this study was to investigate the role of skin secretions in limiting *Bd* infection in captive-bred *A. zeteki*. To do so, we experimentally depleted the skin secretions from *A. zeteki* using dose–response treatments of NE and then measured the inhibitory effectiveness of these secretions against *Bd in vitro*. We then compared pathogen loads and survival of frogs with reduced skin secretions to control frogs when infected with *Bd*. Given the role of skin secretions in protecting against *Bd* in other species of amphibians, we hypothesized that *A. zeteki* skin secretions would be protective against *Bd*. We predicted that the group of *A. zeteki* with reduced skin secretions would have the highest pathogen loads and succumb to disease more quickly than groups of *A. zeteki* with intact skin secretions. Understanding the role of skin secretions in protecting *A. zeteki* from infection is a crucial step towards understanding *Bd* pathogenesis and making well-informed management decisions in this highly endangered amphibian species.

## Methods

### Animal husbandry

We received adult *A. zeteki* frogs from a captive breeding program for this species at the Omaha Zoo (Omaha, NB, USA). None of the frogs had ever been exposed to *Bd* and tested negative for *Bd* upon arrival. We housed frogs individually in plastic containers (19 cm x 11 cm x 14 cm), containing a halved PVC pipe as a hide, and placed the containers on an incline to allow 100 mL of water to pool at one end. We placed containers side by side on a rack (separated using plastic sheeting) in a laboratory maintained at 17.6–20.2°C with a 12:12 light:dark photoperiod. We changed container water and fed the frogs vitamin dusted crickets *ad libitum* three times per week.

### Reduction of skin secretions

Prior to *Bd* exposure, we randomly separated frogs into the four treatment groups. We used norepinephrine (NE) to stimulate the frog's sympathetic nervous system, causing them to release skin secretions from their cutaneous granular glands (Rollins-Smith et al. 2005). NE is eliminated from the body within minutes (Smith and Maani 2019), which allowed us to experimentally reduce skin secretions with few long-term effects on the immune system. Following NE administration, skin secretions typically remain depleted for 21–60 days, after which they are naturally replenished (Rollins-Smith et al. 2005; Ramsey et al. 2010; Pask et al. 2013). Furthermore, release of skin secretions is dose-dependent (Rollins-Smith et al. 2005). Therefore, we used two different doses (high: 40 nmol per gram body mass and low: 10 nmol per gram body mass) of NE to control the amount of skin secretions that were released in each treatment group (Rosa et al., in press).

Specifically, we administered the high dose of NE to the first group of frogs, hereafter the “reduced” group (*N* = 9), and the low dose to the second group of frogs, hereafter the “partially reduced” group (*N* = 9). We gave two control treatment groups injections with sterile amphibian phosphate buffered saline [aPBS, 6.6g/L sodium chloride, 1.15 g/L sodium phosphate, and 0.2 g/L potassium phosphate in 1 L of distilled water, filter sterilized using a sterile nylon mesh filter (Whatman, Little Charlfont, Buckinghamshire, UK)], which did not activate the release of skin secretions. We exposed one of the two control groups, hereafter the “saline” group (*N* = 8) to *Bd*. We exposed the second control group, hereafter “negative control” (*N* = 8), to a sham exposure solution with heat killed *Bd* zoospores rather than live *Bd*.

To administer treatments, we gave frogs subcutaneous injections on the dorsal side, caudal to head and front forelimbs using an EasyTouch 12.7 mm syringe (Vetmed, Fairfield, OH, USA). Following injection with either NE or saline, we placed each frog into a separate, sterile Whirlpak (Whirlpak, Madison, WI, USA) containing 50 mL of HPLC grade water for 15 min, while skin secretions accumulated in the water baths. After this time, we removed the frog and placed it into a clean housing container. To inactivate any endogenous peptidases in the secretion mixture, we acidified the container with AMPs to a final concentration of 1% by volume hydrochloric acid (Woodhams et al. 2006).

To quantify the total secretions released by each frog, we sublimated the samples using a lyophilizer (Labconco, Kansas City, MO, USA). We reconstituted the peptides in 1 mL HPLC grade water and then quantified the amount of material using a standard bicinchoninic acid assay (Micro BCA, ThermoScientific, Rockford, IL, USA) following the manufacturer's instructions, except that we used bradykinin (RPPGFSPFR; Sigma Chemical, St. Louis, MO, USA) to generate a standard curve (Rollins-Smith et al. 2002; Woodhams et al. 2006). We used a microplate reader (BioTek Instruments, Winooski, VT, USA) to quantify the OD of each sample, using a 570 nm wavelength.

### Inhibitory effectiveness of skin secretions against *Batrachochytrium dendrobatidis*

We conducted a *Bd* growth challenge assay to determine the inhibitory effectiveness of the skin secretions collected from each frog. We chose the *Bd* isolate Rio Maria, originally isolated from a *Pristimantis cruentus* frog in Cocle, Panama, because this isolate was pathogenic to *Atelopus* frogs in previous inoculation experiments (Voyles et al. 2018). We cultured the *Bd* on nutrient rich, tryptone agar plates (TGhL; 16 g tryptone, 4 g gelatin hydrolysate, 2 g lactose with 10 g bacteriological agar per 1 L distilled water) at 21°C. Once the cultures reached peak zoospore densities, as observed with light microscopy, we flooded the plates with 3 mL TGhL media for 20 min to collect free swimming zoospores, and then filtered the sample to remove any remaining sporangia (Voyles 2011). We counted *Bd* zoospores using a hemocytometer and then diluted the zoospores using TGhL media to a final concentration of 5 × 10^5^ /mL zoospores.

To set up the experimental plates, we added 40 μL skin secretions collected from each frog with 50 μL of TGhL media, and either 10 μL of live *Bd* to the sample wells or 10 μL of the same concentration of heat killed *Bd* (incubated at 60°C for 10 min) to provide a negative control for *Bd* growth. To provide a negative control for *Bd* inhibition, we combined 10 μL of *Bd* with 20 μL of sterile HPLC grade water. To provide a positive control for *Bd* inhibition, we included 40 μL of skin secretions from *Lithobates pipiens* (a frog species that is known to have skin secretions that are highly inhibitory against *Bd*, L. Reinhart and L. Rollins-Smith, pers. comm. We monitored the plate daily until the day of peak zoospore release. We then used a MTT viability assay and measured OD at 570 nm using a microplate reader to compare *Bd* viability following exposure to skin secretions among treatment groups (Lindauer et al. 2019). We normalized the OD data by subtracting the heat killed negative control wells from our sample and positive control wells.

### *Batrachochytrium dendrobatidis* exposure and survivorship experiment

We weighed each frog to the nearest 0.1 g, and measured snout-vent length (SVL) to the nearest 0.1 mm to calculate frog body condition (mass/SVL) (Dodd 2010; Whiteman et al. 2012). To quantify *Bd* load before beginning the infection experiment, we swabbed each frog with a med-wire swab (MWE, Wiltshire, England, UK) 10 times on the ventral side, 10 times on each thigh, and 5 times on each hand and foot (Hyatt et al. 2007). We repeated these measurements of frog mass, SVL, and collected diagnostic swab samples to determine pathogen load weekly until the end of the experiment.

We cultured the *Bd* isolate Rio Maria and visually monitored the flasks each day using an inverted microscope to determine the day of peak zoospore release, at which point we harvested the liquid *Bd* cultures by passing the culture through sterile filter paper to remove zoosporangia (Voyles 2011). We counted *Bd* zoospores using a hemocytometer and then we diluted the zoospores using TGhL media until they reached a concentration of 6.5 × 10^4^ zoospores /mL. We then placed frogs into the exposure containers (7.6 cm diameter, 3.5 cm height) containing 11 mL of 20% Holtfretter's solution [250 mL, (in mMol) 6.0 NaCl, 0.06 KCL, 0.09, CaCl2, 0.24, NaCO3, pH 7.0; Wright and Whitaker 2001). For the *Bd* exposed frogs, we added 1 mL of live *Bd* culture to the exposure container. For the negative control group, we added 1 mL of heat killed *Bd* culture. The frogs remained in the exposure containers for 24 h, after which we placed them back into their housing containers with 100 mL of 20% Holtfretter's solution.

Following exposures, we monitored frogs twice daily for clinical signs of disease (Voyles et al. 2009). Once frogs began showing clinical signs, such as abnormal posture, cutaneous erythema, lethargy, loss of righting reflex, and irregular skin sloughing (Voyles et al. 2009), we collected one final diagnostic *Bd* swab before euthanizing them via shallow immersion in a bath of 0.1% MS-222 (tricaine methanesulfonate, Fisher Scientific, Ferndale, WA, USA) and placed them in a tube with formalin for preservation. For the uninfected control frogs, we collected swabs weekly for two weeks following the date of the death of the last *Bd*-exposed frogs, collected a final swab for *Bd* diagnostic analysis, and then terminated the experiment.

### DNA extraction and qPCR amplification

To extract *Bd* DNA from diagnostic swabs, we used a DNeasy Blood and Tissue DNA Extraction Kit following the manufacturer's directions (Qiagen, Valencia, CA, USA; animal tissue protocol). We used real time quantitative polymerase chain reaction (qPCR) to quantify *Bd* load for each swab (Boyle et al. 2004). We ran the assay on a QuantStudio 3 Real-Time PCR instrument (Life Technologies, Singapore). We analyzed samples in duplicate with an internal positive control (IPC, Garland et al. 2010) and a dilution set of plasmid standards (Pisces Molecular, Boulder, CO, USA) to quantify *Bd* load. We converted plasmid copy numbers to genomic equivalents using the formula *Bd* load = quantity*40. If one of the duplicate samples appeared positive, we checked the cycle threshold (*C_t_*) value to determine if low level infection was likely and verified that the qPCR was working properly by confirming IPC amplification.

### Statistical analyses

We performed all analyses using R version 4.0.2 (R Core Team 2020). The data on the quantity of skin secretions recovered from each treatment group following injection with either NE or saline were not normally distributed with transformations. Therefore, we used a Kruskal–Wallis test to analyze these data. We followed this analysis with a pairwise Wilcox test, using a Bonferroni correction, to determine if there were differences in the amounts of skin secretions collected among treatment groups. We tested the inhibitory effectiveness of the skin secretions against *Bd* by comparing growth when combined with skin secretions for all groups. We combined the two groups that received injections of sterile saline into one “saline” group for analysis. We used a one-way analysis of variance (ANOVA) and Tukey HSD *post hoc* tests to compare *Bd* growth and determined the inhibitory effectiveness of skin secretions against *Bd* across treatment groups.

We calculated change in body condition by subtracting the final body condition from the initial body condition for each frog. To evaluate differences in initial body condition among the treatment groups at the start of the experiment, as well as differences in body condition between the start and end of the experiment, we used ANOVA with Tukey HSD *post hoc* tests. To compare body condition across treatment groups, we ran a linear mixed-effects model (LMM) with body condition as the dependent variable, treatment group and time as interacting fixed effects, and individual frogs as a random effect (package: “nlme,” function: “lme,” Pinheiro, DebRoy, Sarkar, & Team 2022).

To see how *Bd* load changed over time, we ran a GLMM with a Poisson distribution with *Bd* load as the dependent variable, treatment group and time as interacting fixed effects and individual frogs as the random effect (package: “lme4,” function: “glmer,” Bates, Maechler, Bolker, Walker 2015). We used an ANOVA followed by Tukey HSD *post hoc* tests to compare mean survival time as well as *Bd* load, presented as the mean of genomic equivalents, on the day of death among the three *Bd* exposed treatment groups. Using the R package “survival” (Therneau and Gramsch, 2000), we generated a Kaplan–Meier survival curve and analyzed survival data using a log rank test.

## Results

We found that the quantity of secretions released differed significantly among the reduced, partially reduced, and saline treatment groups (Kruskal–Wallis, *Χ*^2^ (2) = 9.413, *P* = 0.009; Fig. 1B). We grew *Bd* in the presence of the skin secretions to compare inhibitory effectiveness of the skin secretions across treatment groups. There was significantly less *Bd* growth in the wells containing *L. pipiens* secretions (which we included as a positive control for *Bd* inhibition) compared to all other treatment groups with skin secretions from *A. zeteki* (ANOVA: *F*_4_,_74_ = 15.96, *P* < 0.001; Tukey *HSD, P* < 0.001; Fig. 1C). We found significantly increased *Bd* growth in the samples collected from the reduced group compared to both the positive control group (Tukey HSD, *P* < 0.02; Fig. 1C) and the saline group (Tukey HSD, *P* < 0.01; Fig. 1C).

**Fig. 2:**
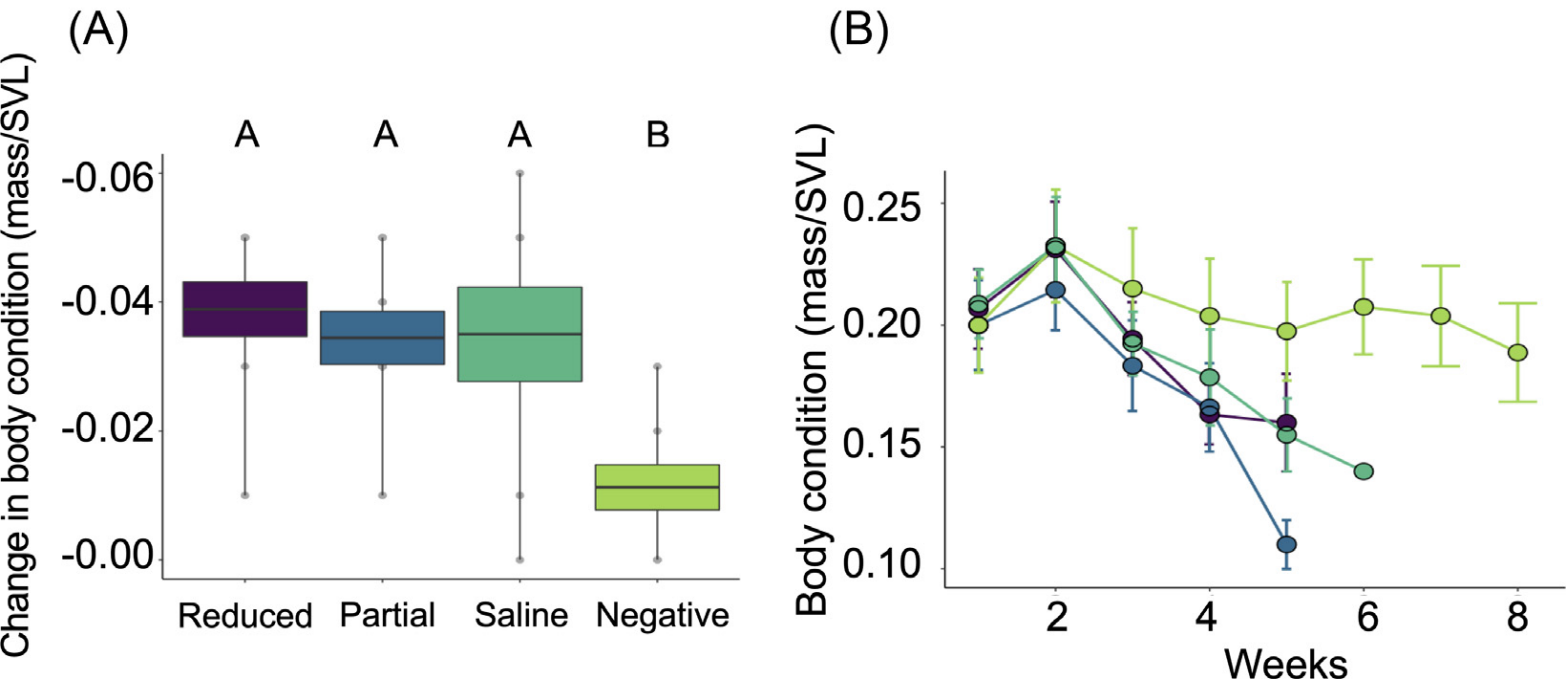
**(A)** The mean change in body condition (mass/SVL) in groups of *A. zeteki* frogs that had their skin secretions reduced (purple), partially reduced (blue), intact (with a saline treatment; teal), and were subsequently exposed to *Bd* or heat-killed *Bd* (negative control; green). **(B)** Body condition was measured weekly until the end of the experiment for all five treatment groups. Error bars represent standard error of the mean. Letters indicate significant differences among groups at the level of *P* = 0.05

The body conditions of frogs in all four groups were not significantly different at the start of the experiment (ANOVA: *F*_3_,_30_ = 0.068, *P* = 0.977). However, in comparing the body condition of the frogs among groups, we found that the *Bd* exposed treatment groups had significantly lower mean body conditions from the beginning to the end of the experiment compared to the negative control group ​​(ANOVA: *F*_3_,_30_ = 6.289, *P* < 0.001; Tukey HSD, *P* < 0.05; Fig. 2A). There were no significant differences in the change in body condition among the three *Bd* exposed groups at the end of the experiment (ANOVA: *F*_2_,_23_ = 0.218, *P* = 0.806; Fig. 2A). Additionally, there was no significant difference in the effect of treatment group and time on body condition among the *Bd*-exposed groups (LMM, *F* = 0.2519, *P* = 0.8; Fig. 2B).

**Fig. 3:**
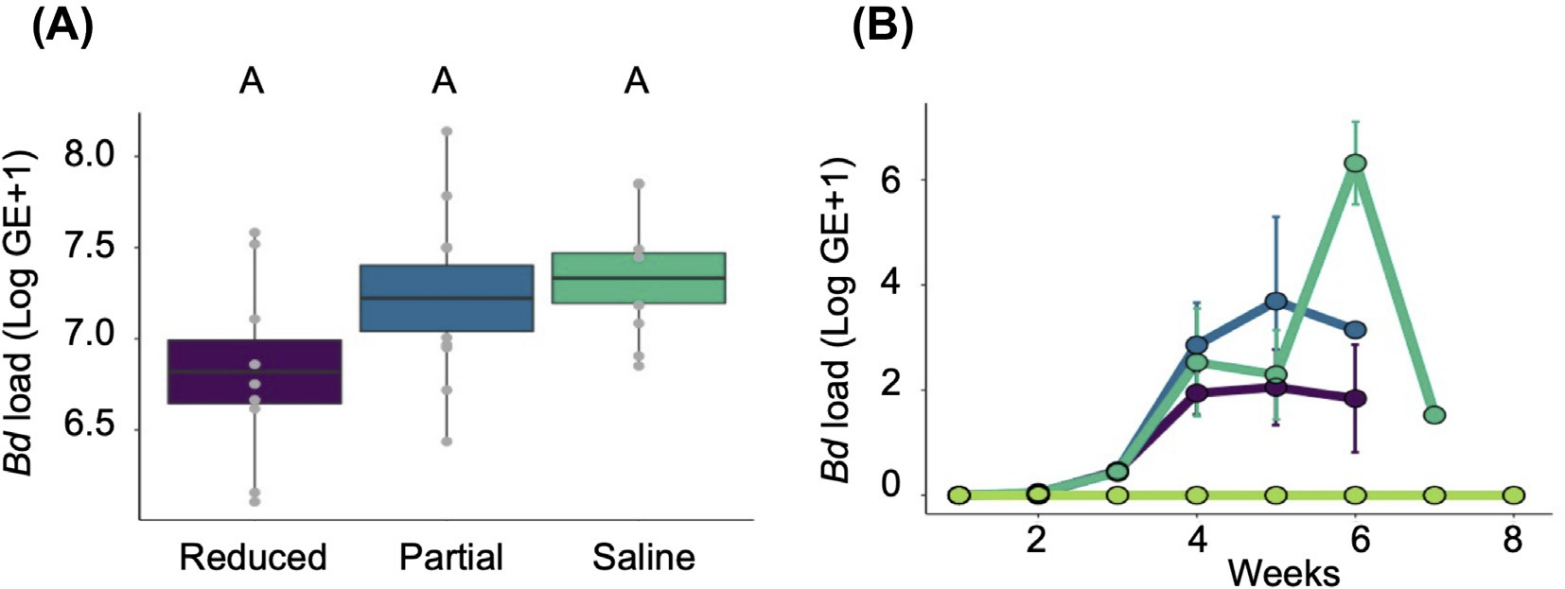
**(A)** The mean infection intensity (±SE) of *Bd* on skin swab samples that were collected on the date of death for groups of *A. zeteki* frogs that had their skin secretions reduced (purple), partially reduced (blue), intact (with a saline treatment; teal). Infection intensity was calculated as log (genomic equivalents + 1) following qPCR analysis. **(B)** Change in mean infection intensity (±SE) of *Bd* over time for all four treatment groups. Letters indicate significant differences among groups at the level of *P* = 0.05

The *Bd* isolate Rio Maria was highly pathogenic in all groups of exposed *A. zeteki* frogs. All *Bd* exposed frogs tested positive with qPCR and *Bd* infection prevalence was 100% for all three exposed treatment groups at each post-exposure time point (when we collected diagnostic samples) and at the time of death. Although there were no significant differences of *Bd* load on the date of death among exposed groups (ANOVA: *F*_2_,_23_ = 2.61, *P* = 0.0951; Fig. 3A), there was a trend in the data suggesting that the saline group had slightly higher infection intensities, followed by the partially reduced and reduced groups respectively. There was not a significant difference in the effect of treatment group and time on the pathogen load among the *Bd*-exposed groups (GLMM with a Poisson distribution, *Χ*^2^ = 0.9435, *df* = 2, *P* = 0.6; Fig. 3B). None of the frogs in the negative control group showed any signs of *Bd* infection by qPCR at the start and end of the experiment. All but one of the negative control frogs showed weakly positive qPCR results towards the middle of the experiment, prompting a rerun of the qPCR to validate this finding. Because the same individual frogs tested negative with subsequent testing and never showed any clinical signs of infection, we believe these were likely false positives (possibly due to sample contamination during either the swabbing or extraction steps). Contamination may have occurred because we used ethanol to disinfect measuring instruments (e.g., calipers) during swabbing and ethanol kills *Bd* but does not degrade its DNA (Lindauer and Voyles 2019).

**Fig. 4:**
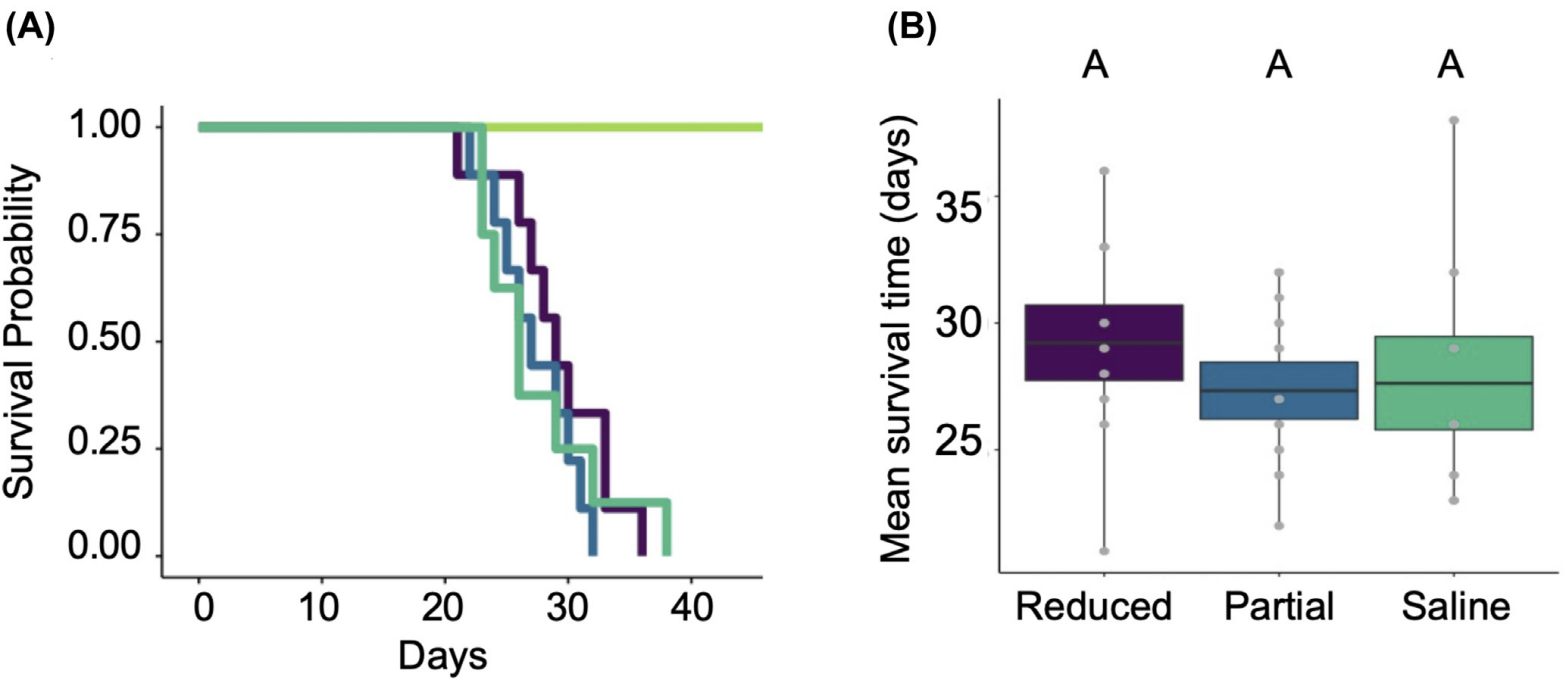
(**A**) Survivorship after exposure to *Bd* in groups of *A. zeteki* frogs that had their skin secretions reduced (purple), partially reduced (blue), intact (with a saline treatment; teal) or that were exposed to heat killed *Bd* (negative control; green). (**B**) The mean survival time (±SE) of the reduced (purple), partially reduced (blue), and saline (teal) treatment groups following exposure to *Bd*. Letters indicate significant differences among groups at the level of *P* = 0.05

All *Bd*-inoculated frogs became infected, developed clinical signs of chytridiomycosis, and died within six weeks of exposure. None of the negative control frogs developed clinical signs of chytridiomycosis, all survived past the end of the experiment, and all were *Bd* negative two weeks after the termination of the experiment. There were no significant differences in survival results among the three *Bd* exposed treatment groups, regardless of skin secretion status (Kaplan–Meier, *Χ*^2^ = 1.2, *df* = 2, *P* = 0.6; Fig. 4A). Mean survival time among the three *Bd* exposed groups was also not significantly different (ANOVA: *F*_2_,_23_ = 0.48, *P* = 0.625; Fig. 4B).

## Discussion

Populations of golden frogs (*A. zeteki and A. varius*) experienced severe declines when *Bd* emerged in Panama (La Marca et al. 2005; Lips et al. 2006; Crawford et al. 2010). Captive breeding programs have ensured the continued survival of *A. zeteki*, albeit in *ex situ* conditions (Lewis et al. 2019). Given that *Bd* is still present and highly pathogenic in Panama (Perez et al. 2014; Voyles et al. 2018; Rosa et al., in press), reintroduction efforts of captive *Atelopus* hinge on expanding our understanding the host–pathogen interactions of *A. zeteki* with *Bd*. Skin secretions are an innate immune component that are thought to be important for protecting many amphibian species from *Bd* (Rollins-Smith 2009; Varga et al. 2019). The purpose of this study was to investigate the importance of skin secretions in protecting *A. zeteki* from chytridiomycosis. We used NE to experimentally reduce captive-reared frogs of their skin secretions in a dose dependent manner. We tested the inhibitory effectiveness of the secretions against *Bd in vitro* and then exposed frogs that had their skin secretions experimentally reduced to *Bd*. We predicted that frogs with reduced and partially reduced secretions would experience a greater change in body condition, higher pathogen loads, and decreased survival compared to frogs with intact secretions.

Our results did not support our hypothesis and instead suggest that captive-bred *A. zeteki* skin secretions increased *Bd* growth *in vitro* and may have led to slightly higher infection intensities in controlled exposure experiments. Our *in vitro* assays showed that the skin secretions collected from the reduced, partially reduced, and saline frog groups were poor *Bd* inhibitors, unlike our control secretions from *L. pipiens*. Rather, we found that the skin secretions enhanced *Bd* growth *in vitro*. Specifically, *Bd* growth was significantly higher in wells containing skin secretions from the reduced group compared to both the positive control wells (containing *Bd* and TGhL media) and to the saline group wells (containing *Bd* and skin secretions from the saline groups). These findings were further supported by the infection and mortality patterns that we observed in our *in vivo* experiment; the frogs in the reduced group had lower *Bd* loads and survived slightly longer than the frogs in the saline (control) frogs that maintained their skin secretions. While it is possible that NE treatment could have led to unintended immunosuppressive effects (e.g., impairment of adaptive immune functioning; Rollins-Smith 2020), we suggest that it is unlikely given that NE is eliminated from the body in minutes (Smith and Maani 2019) and reduces stress hormones such as corticosterone and aldosterone, which are known to suppress lymphocyte function (Morra et al. 1990). Taken together, our findings add to our understanding of the considerable complexities of amphibian host responses to *Bd*. We suggest that it will be key for investigators to recognize that the role of skin secretions in protecting amphibians may be individual-, population-, and/or species-specific.

These results, while surprising, fit within a broader context of the diversity of host responses to *Bd* infection. With respect to skin secretions, many amphibian species produce skin secretions that are capable of inhibiting *Bd* growth *in vitro*, but still appear to be highly susceptible to chytridiomycosis and have experienced *Bd*-induced population declines (e.g., *Agalychnis lemur*; Lips et al. 2006; Woodhams et al. 2006, 2008; Crawford et al. 2010). However, some species exhibit resistance to infection in the wild, but *in vitro* testing has shown that their skin secretions are virtually ineffective at inhibiting *Bd* (Conlon 2011; Woodhams et al. 2016). One potential explanation for our results is that some components of the chemically rich skin secretions enhanced pathogen growth despite the presence of other inhibitory immune components. Besides AMPs, skin secretions also contain proteins, carbohydrates, sugars, and biomolecules with functions other than immune defense, which may serve as nutrients or chemical signals for *Bd* (Moss et al. 2008; Van Rooji et al. 2015; Varga et al. 2019; Mayer et al. 2021; Wang et al. 2021). Another potential explanation is that the immunogenic protection of the skin secretions stems from interactions with other components of the immune system, and by isolating the skin secretions *in vitro*, they lose their inhibitory effectiveness (Conlon 2011). For example, peptides isolated from *Rana sierrae* were not inhibitory towards *Bd* on their own, but it did facilitate the growth of inhibitory microbes, such as *Janthinobacterium lividum* in *Rana sierrea* (Woodhams et al. 2020).

In addition to skin secretions, a wide range of additional host defensive factors are likely involved in protection against severe chytridiomycosis (Harris et al. 2006; Rollins-Smith et al. 2011; Savage and Zamudio 2011; McMahon et al. 2014). For example, the skin microbiome (and beneficial cutaneous bacteria in particular) may mediate susceptibility (Harris et al. 2006; Rollins-Smith et al. 2011). Furthermore, although previous studies suggested amphibians lack a robust adaptive immune defense against *Bd*, immunoglobulins (IgM and IgY) are known to neutralize *Bd in vitro* (Ramsey et al. 2010; Rollins-Smith et al. 2011), splenocyte abundance may increase with multiple *Bd* exposures (McMahon et al. 2014), and the complement system may be initiated in early stages of infection (Grogan et al. 2018). Furthermore, one study has suggested host behavioral avoidance of *Bd* may also be an important defensive strategy for some amphibian species (McMahon et al. 2014). Ultimately, a species’ defensive capacity against *Bd* will be most completely defined when we can investigate a composite of a wide range of diverse protective traits.

Additionally, it is important to consider how host–pathogen interactions may shift over time (Voyles et al. 2018; Rothstein et al. 2021). Previous studies comparing the inhibitory effectiveness of skin secretions from captive and wild *A. varius* showed that secretions collected from persisting populations have a much higher inhibitory effectiveness against *Bd* (Voyles et al. 2018). This finding suggests the intriguing possibility that *A. varius* that survived the initial selective sweep of epizootic events may have evolved more effective skin secretion defenses compared to their counterparts that were moved into captivity and remained *Bd* naïve (as were the frogs in this study). The possibility of adaptive changes in host immunity over time is also supported by preliminary evidence of immunogenetic traits (increased heterozygocity in MHC loci) that are associated with resistance to *Bd* in a North American frog species (*Lithobates yavapaiensis*, Savage and Zamudio 2011). While the possibility of evolutionary shifts in host responses remains to be fully investigated in *A. varius*, as well as additional host species, the prospect of generating more inhibitory skin secretions following initial disease outbreaks may help explain how some populations of *A. varius* are persisting and rebounding in areas of Central America where they previously experienced disease-induced declines (González-Maya et al. 2013, 2018; Voyles et al. 2018).

Our results also have important implications for amphibian conservation planning. One central goal of the “Project Golden Frog” captive breeding program is to repatriate and reintroduce *A. zeteki* back into the wild (Gagliardo et al. 2008). Yet, our results confirm that captive populations of *A. zeteki* remain highly susceptible to *Bd*, which may render release of susceptible individuals into the wild an inadvisable first step (Lewis et al. 2019). Several strategies have been attempted to enhance or augment the effectiveness of immunity in captive *A. zeteki* populations. For example, as the microbiome is important in defending against *Bd* in many species (Rebollar et al. 2020), some efforts have been directed to use probiotic application for captive *Atelopus* to test for protection against *Bd* (Becker et al. 2021). However, bioaugmentation attempts to incorporate *Bd*-inhibiting bacteria to the microbiome of *A. zeteki*, have so far been unsuccessful (Becker et al. 2011, 2015,2021). Additionally, genomic studies have shown that while the innate and acquired immune responses of golden frogs are vigorous, they are ultimately ineffective at preventing infection, disease development, and mortality (Ellison et al. 2014, 2015). The current study suggests that skin secretions of *A. zeteki* are not only ineffective at inhibiting *Bd in vitro*, but they may also be enhancing pathogen growth *in vivo* and thereby leading to increased disease and mortality. Thus, we suggest that continued investigation into the host–pathogen interactions of captive *A. zeteki* and *Bd* is warranted to unravel which components of the immune system should be focal points for investigation prior to initiating reintroduction efforts.

Both intraspecific and interspecific variation in host immune response to the same pathogen can result in vastly different infection outcomes, which is a major focus of ecoimmunologists (Schoenle, Downs, and Martin 2018). We provide evidence that the skin secretions of captive *A. zeteki* do not protect frogs from chytridiomycosis and may instead be enhancing *Bd* growth, leading to greater susceptibility to chytridiomycosis. These results are unexpected given that skin secretions are a ubiquitous and important innate immune component across vertebrate phyla (Boparai and Sharma 2020). They also underscore that some immune components that are effective in some species cannot be generalized across all host species or even for the same species at different time points. This is an important consideration in the field of ecoimmunology, where implementing conservation strategies depends on in-depth knowledge of immunity in both closely and distantly related species. Given the severity of disease induced biodiversity loss, additional research on host immune heterogeneity is urgently needed to understand the host–pathogen interactions, especially in animals impacted by EIDs.

## Animal ethics

We performed all experiments with approval from, and in accordance with, the ethical standards of the UNR Institutional Animal Care and Use Committee under protocol #20–08–1063.

## Data Availability

The data underlying this article will be shared on reasonable request to the corresponding author.

## Notes

From the symposium “Ecoimmunology: what unconventional organisms tell us after two decades” presented at the annual meeting of the Society for Integrative and Comparative Biology virtual annual meeting, January 3–February 28, 2022.
